# Anemia predicts poor outcomes of COVID-19 in hospitalized patients: a prospective study in Iran

**DOI:** 10.1186/s12879-021-05868-4

**Published:** 2021-02-10

**Authors:** Masood Faghih Dinevari, Mohammad Hossein Somi, Elham Sadeghi Majd, Mahdieh Abbasalizad Farhangi, Zeinab Nikniaz

**Affiliations:** 1 Liver and Gastrointestinal Diseases Research Center, Tabriz University of Medical Sciences, Tabriz, Iran; 2 Student Research Committee, Tabriz University of Medical Sciences, Tabriz, Iran; 3 Community Nutrition Department, Tabriz University of Medical Sciences, Tabriz, Iran

**Keywords:** COVID-19, Anemia, Mortality, Ventilator requirement, ICU admission

## Abstract

**Background:**

There are limited number of studies with controversial findings regarding the association between anemia at admission and coronavirus disease 2019 (COVID-19) outcomes. Therefore, in this research, we aimed to investigate the prospective association between anemia and COVID-19 outcomes in hospitalized patients in Iran.

**Methods:**

In this prospective study, the data of 1274 consecutive patients hospitalized due to COVID-19 were statistically analyzed. All biomarkers, including hemoglobin and high-sensitivity C-reactive protein (hs-CRP) levels were measured using standard methods. Anemia was defined as a hemoglobin (Hb) concentration of less than 13 g/dL and 12 g/dL in males and females, respectively. Assessing the association between anemia and COVID-19 survival in hospitalized patients was our primary endpoint.

**Results:**

The mean age of the participants was 64.43 ± 17.16 years, out of whom 615 (48.27%) were anemic subjects. Patients with anemia were significantly older (*P* = 0.02) and had a higher frequency of cardiovascular diseases, hypertension, kidney disease, diabetes, and cancer (*P* < 0.05). The frequency of death (anemic: 23.9% vs. nonanemic: 13.8%), ICU admission (anemic: 27.8% vs. nonanemic:14.71%), and ventilator requirement (anemic: 35.93% vs. nonanemic: 20.63%) were significantly higher in anemic patients than in nonanemic patients (*P* < 0.001). According to the results of regression analysis, after adjusting for significant covariate in the univariable model, anemia was independently associated with mortality (OR: 1.68, 95% CI: 1.10, 2.57, *P* = 0.01), ventilator requirement (OR: 1.74, 95% CI: 1.19, 2.54, *P* = 0.004), and the risk of ICU admission (OR: 2.06, 95% CI: 1.46, 2.90, *P* < 0.001).

**Conclusion:**

The prevalence of anemia in hospitalized patients with COVID-19 was high and was associated with poor outcomes of COVID-19.

## Background

Severe acute respiratory syndrome coronavirus (SARS-COV-2), known as COVID-19, is the new type of coronavirus responsible for the latest pandemic in the world [1]. This virus was initially identified in December 2019 in patients with flu-like syndrome and pneumonia in Wuhan, China, and it is rapidly spreading worldwide [1].

COVID-19 is mainly characterized by respiratory symptoms, but it has variable degrees of severity, from mild upper respiratory illness to severe interstitial pneumonia and multiorgan failure leading to death [2]. There are different clinical characteristics and comorbidities associated with severity, hospitalization, and mortality in COVID-19, such as older age, male sex, and severe obesity [3].

There are different laboratory findings upon admission that are early predictors of COVID-19 patients. Patients with severe disease had significantly increased white blood cell (WBC) counts and decreased lymphocyte and platelet counts. Biomarkers of inflammation, cardiac, liver, kidney function, and coagulation markers were also elevated in these patients [4]. In COVID-19 patients, inflammation can lead to an alternation of iron hemostasis and reduced intestinal iron absorption, resulting in the reduced availability of the metal for erythropoiesis and the production of hemoglobin (Hb) [5]. In this regard, some studies focused on the association between anemia and the severity or mortality of COVID-19, the results of which were controversial. Several studies, mostly conducted in China, showed that anemic patients were more likely to have severe disease and higher mortality [5–8]. However, in a study in Italy, Cecconi et al. did not observe any association between anemia and poor outcomes of COVID-19 (8). Similarly, in a study in China, Yang et al. reported no association between low Hb levels and COVID-19 outcomes in hospitalized patients [9].

Currently, COVID-19 is a public health emergency of international concern and there are limited studies with controversial findings regarding the association between anemia at admission and COVID-19 outcomes. Accordingly, in this study, we aimed to investigate the prospective association between anemia and COVID-19 outcomes in hospitalized patients in Tabriz, Iran.

## Methods

In this prospective study, the data of the AzarCoRe (East Azar COVID-19 Registry) were used. In this registry, the patients were registered prospectively based on reverse transcription-polymerase chain reaction (RT-PCR) results or lung imaging features.

The demographic, clinical laboratory, and anthropometric data were collected using questionnaires by trained nurses in all COVID-19-related units. All patients were followed-up until they were discharged from the hospital or death.

All biomarkers, including Hb and high-sensitivity C-reactive protein (hs-CRP) levels were measured in the laboratory of Imam Reza Hospital of Tabriz, Iran using standard methods.

We registered 1406 patients with COVID-19 in the AzarCoRe. After excluding patients with incomplete information, the data of 1274 patients were statistically analyzed.

### Outcomes

The main obejective of this study was to evaluate the association between anemia and COVID-19 survival in hospitalized patients. In addition, we analyzed the association between anemia and the probability of ICU admission and the requirement of mechanical ventilation at any point.

Anemia was defined according to the World Health Organization (WHO) definition as Hb concentration of less than 13 g/dL and 12 g/dL in males and females, respectively [10].

Disease severity was defined based on the quick sequential organ failure assessment (qSOFA) score and confusion, urea, respiratory rate, blood pressure, and 65 years of age or older (CURB-65) score. The qSOFA was calculated by summing the scores of the following criteria: Glasgow Coma Scale < 15, respiratory rate (RR) ≥22, and systolic blood pressure (SBP) ≤100. Curb-65 was calculated as the sum of the following findings, each of which obtained one point: Glasgow Coma Scale < 15, blood urea nitrogen > 19 mg/dL, RR ≥30, SBP < 90 mmHg or diastolic blood pressure (DBP) ≤60 mmHg, and age ≥ 65 years. Patients with qSOFA scores ≥2 or CURB-65 scores ≥3 were considered as severe COVID-19 cases [11, 12].

### Statistical analysis

For statistical analysis, SPSS version 25.0 (IBM Corporation, NY, USA) was used. The normality of the data distribution was analyzed by the Kolmogorov-Smirnov test. The quantitative and qualitative values were reported as the mean (SD) and frequency (%), respectively. Between-group comparisons were performed using the independent t-test for continuous variables and the chi-square test for categorical variables. Logistic regression was used to analyze the association between anemia and COVID-19 outcomes in univariable and multivariable models. Factors that had a significant association in the univariable model were considered in the multivariable model. For all analyses, a *P*-value< 0.05 was considered significant.

## Results

In the present study, 1274 COVID-19 patients with a mean age of 64.43 ± 17.16 years were included, out of whom 615 (48.27%) were anemic. There was a significant association between sex and anemia (*P* = 0.03). As shown in Table 1, patients with anemia were significantly older (*P* = 0.02) and had a higher frequency of cardiovascular diseases (*P* = 0.03), hypertension (*P* < 0.001), kidney diseases (*P* < 0.001), diabetes (*P* < 0.001), and cancer (*P* < 0.001). Moreover, there was a significant association between body mass index (BMI) and anemia (*P* = 0.02).

**Table 1:** The demographic characteristics of patients with COVID-19

Demographic variables	Total (***n*** = 1274)	No-Anemia (***n*** = 659)	Anemia (***n*** = 615)	***p***-value
**Age (years) Mean ± SD**	64.43 ± 17.16	62.39 ± 17.15	64.53 ± 17.12	0.02
**Sex,**				
**Males n (%)**	706 (55.41)	384 (58.27)	322 (52.35)	0.03
**Females n (%)**	568 (44.59)	275 (41.73)	293 (47.65)	
**Smoking n (%)**	74 (5.80)	40 (6.06)	34 (5.52)	0.74
**Comorbidities n (%)**				
**CVD**	435 (34.14)	138 (20.94)	297 (48.29)	0.03
**Respiratory diseases**	171 (13.42)	89 (13.50)	82 (13.33)	0.9
**HTN**	504 (39.56)	244 (37.02)	260 (42.27)	0.03
**Kidney diseases**	112 (8.79)	26 (3.94)	86 (13.98)	< 0.001
**Diabetes**	288 (22.60)	121 (18.36)	167 (27.15)	< 0.001
**Carcinoma**	59 (4.63)	11 (1.66)	48 (7.80)	< 0.001
**Liver diseases**	24 (1.88)	13 (1.97)	11 (1.78)	0.85
**Autoimmune diseases**	14 (1.09)	4 (0.6)	10 (1.62)	0.07
**BMI categories n (%)**				0.02
**Underweight (BMI < 18.5 kg/m**^**2**^**)**	420 (32.96)	196 (29.7)	224 (36.4)	
**Normal weight (BMI: 18.5–25 kg/m**^**2**^**)**	528 (41.44)	287 (43.5)	241 (39.1)	
**Overweight/Obese (BMI > 25 kg/m**^**2**^**)**	326 (25.5)	176 (26.7)	150 (24.3)	
**hs-CRP (mg/l)**	4.01 ± 8.61	4.15 ± 9.77	3.85 ± 7.29	0.84
**Severe disease n (%)**	67 (5.25)	23 (3.49)	38 (6.17)	0.01
**Temperature (°C)**	36.83 ± 4.13	36.92 ± 3.55	36.67 ± 4.91	0.54
**Hypoxia n (%)**	466 (36.57)	242 (36.72)	224 (36.42)	0.81

*CVD* Cardiovascular disease, *HTN* Hypertension, *BMI* Body mass index, *hs-CRP* high sensitive C-Reactive protein

*p-value of independent t-test

**p-value of chi-square test

**Fig. 1:**
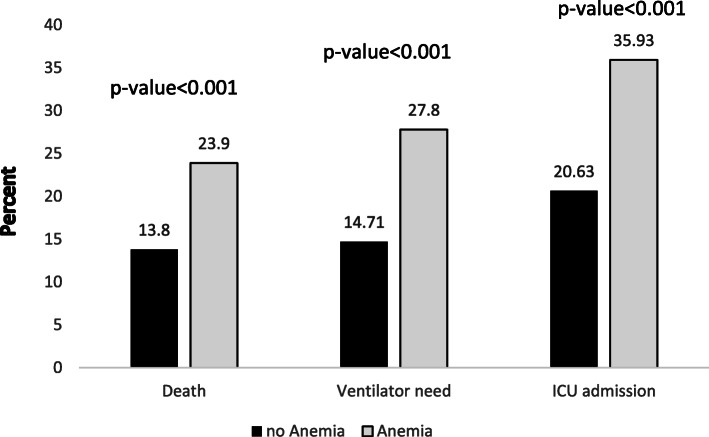
The frequency of outcomes of COVID-19 stratified by anemia status

The frequency of COVID-19 outcomes stratified by anemia status is shown in Fig. 1. The frequency of death, ICU admission, and ventilator requirement were significantly higher in anemic patients than in nonanemic ones (*P* < 0.001).

Table 2 depicts the association between anemia (independent factor) and COVID-19 mortality in univariable and multivariable models. In the multivariable model, after adjusting for variables with significant associations in the univariable model such as age, hypoxia, respiratory diseases, diabetes, smoking status, and diseases severity, an independent significant association was observed between anemia (OR: 1.68, 95% CI: 1.10, 2.57, *P* = 0.01) and COVID-19 mortality.

**Table 2:** The logistic regression analysis of anemia and death during hospitalization in patients with COVID-19

Variables	Univariate model			Multivariate model^a^		
	Odds Ratio	CI	*P*-value	Odds Ratio	CI	*P*-value
Age	1.02	1.01, 1.03	< 0.001	1.03	1.01, 1.04	< 0.001
Sex	0.82	0.62, 1.09	0.17			
BMI	1.00	0.97, 1.03	0.98			
Temperature	0.99	0.97, 1.02	0.74			
Hypoxia	3.30	2.45, 4.46	< 0.001	2.30	1.51, 3.50	< 0.001
CVD	1.27	0.92, 1.76	0.13			
Respiratory diseases	1.51	1.02, 2.24	0.03	1.07	0.61, 1.87	0.80
HTN	1.18	0.88, 1.58	0.24			
Kidney diseases	1.27	0.80, 2.01	0.30			
Cancers	1.24	0.66, 2.34	0.49			
Autoimmune diseases	3.16	0.04, 2.41	0.26			
Diabetes	1.60	1.16, 2.19	0. 004	1.52	0.96, 2.41	0.07
Liver diseases	0.20	0.02, 1.52	0.12			
Smoking	2.00	1.18, 3.40	0.01	1.76	0.88, 3.58	0.11
Disease severity	2.45	1.38, 4.34	0.002	2.03	0.96, 2.41	0.06
hs-CRP level	0.96	0.9, 1.01	0.80			
Anemia	2.01	1.50, 2.69	< 0.001	1.68	1.10, 2.57	0.01**

Dependent variable: Death

*CVD* cardiovascular disease, *HTN* Hypertension, *BMI* Body mass index, *CRP* C-Reactive protein, *hs-CRP* high sensitive-C reactive protein

^a^Multivariate model adjusted for all variables with significant association in Univariate model

**adjusted for age, hypoxia, respiratory diseases, diabetes, smoking status, diseases severity

The association between anemia and ventilator requirement in hospitalized patients with COVID-19 is shown in Table 3. After adjusting for variables that had a significant association with ventilator requirement in the univariable model such as age, hypoxia, respiratory diseases, diseases severity, it was verified that anemia was an independent and significant risk factor for ventilator requirement (OR: 1.74, 95% CI: 1.19, 2.54, *P* = 0.004).

**Table 3:** The logistic regression analysis of anemia and mechanical ventilator requirement during hospitalization in patients with COVID-19

Variables	Univariate model			Multivariate model^a^		
	Odds Ratio	CI	*P*-value	Odds Ratio	CI	*P*-value
Age	1.01	1.00, 1.02	0.001	1.01	1.00, 1.02	0.005
Sex	0.78	1.00, 1.02	0.08			
BMI	0.99	0.96, 1.02	0.89			
Temperature	0.97	0.93, 1.01	0.26			
Hypoxia	4.12	3.06, 5.56	< 0.001	3.47	2.37, 5.07	< 0.001
CVD	1.35	0.99, 1.85	0.05			
Respiratory Diseases	1.59	1.09, 2.32	< 0.001	1.45	0.90, 2.34	0.11
HTN	1.25	0.95, 1.66	0.10			
Kidney diseases	1.14	0.72, 1.81	0.56			
Cancers	1.45	0.80, 2.62	0.21			
Autoimmune diseases	0.32	0.03, 2.31	0.25			
Diabetes	1.22	0.89, 1.67	0.21			
Liver diseases	0.35	0.08, 1.52	0.16			
Smoking	1.44	0.81, 2.55	0.20			
Disease severity	3.61	2.09, 6.24	< 0.001	2.73	1.48, 5.03	< 0.001
CRP level	0.99	0.98, 1.01	0.89			
Anemia	2.12	1.60, 2.81	< 0.001	1.74	1.19, 2.54	0.004**

Dependent variable: requirement for mechanical ventilation

*CVD* cardiovascular disease, *HTN* Hypertension, *BMI* Body mass index, *CRP* C-Reactive protein

^a^Multivariate model was adjusted for all variables with significant association in Univariate model

**Adjusted for age, hypoxia, respiratory diseases, diseases severity, and Anemia

As shown in Table 4, in hospitalized patients with COVID-19, after adjusting for sex, hypoxia, smoking status, and disease severity, anemia was an independent and significant risk factor for ICU admission (OR: 2.06, 95% CI: 1.46, 2.90, *P* < 0.001).

**Table 4:** The logistic regression analysis of anemia and ICU admission during hospitalization in patients with COVID-19

Variables	Univariate model			Multivariate model^a^		
	Odds Ratio	CI	*P*-value	Odds Ratio	CI	*P*-value
Age	1.00	0.99, 1.01	0.15			
Sex	0.73	0.57, 0.94	0.01	0.66	0.46, 0.93	0.02
BMI	0.99	0.97, 1.02	0.83			
Temperature	0.97	0.93, 1.01	0.21			
Hypoxia	3.04	2.34, 3.96	< 0.001	2.57	1.84, 3.60	< 0.001
CVD	1.26	0.95, 1.68	0.1			
Respiratory diseases	1.40	0.98, 1.99	0.05			
HTN	1.04	0.81, 1.35	0.72			
Kidney diseases	1.10	0.72, 1.66	0.65			
Cancers	1.46	0.84, 2.54	0.17			
Autoimmune diseases	0.47	0.10, 2.14	0.33			
Diabetes	1.16	0.87, 1.55	0.30			
Liver diseases	0.51	0.17, 1.51	0.22			
Smoking	1.67	1.01, 2.76	0.04	1.06	0.54, 2.08	0.85
Disease severity	2.43	1.41, 4.17	0.001	1.90	1.02, 3.52	0.04
CRP level	0.99	0.97, 1.01	0.47			
Anemia	2.02	1.58, 2.63	< 0.001	2.06	1.46, 2.90	< 0.001**

Dependent variable: ICU admission

*CVD* cardiovascular disease, *HTN* Hypertension, *BMI* Body mass index, *CRP* C-Reactive protein

^a^Multivariate model adjusted for all variables with significant association in Univariate model

**adjusted for sex, hypoxia, smoking status, diseases severity, and Anemia

## Discussion

COVID-19 is an infectious disease with a high mortality rate. In this regard, different studies have attempted to investigate the factors associated with poor outcomes in these patients. Accordingly, in the present study, we assessed the association between anemia status on admission and COVID-19 outcomes. Our results showed that the prevalence of anemia was high (48%) in hospitalized COVID-19 patients. However, Bellmann-Weiller reported that 24.7% of patients with COVID-19 on admission were anemic in Austria [5]. The higher prevalence of anemia in our study may be related to a higher prevalence of pre-existing anemia in Iran, a higher percentage of the female population in our study, and differences in comorbidities, anemia definition used in different studies, and disease severity.

In addition, our results showed that anemic patients were significantly more likely to develop poor outcomes of COVID-19, including death, ventilator need, and ICU admission. This finding is in line with the results of previous studies that reported a significantly lower level of Hb in patients with severe COVID-19 disease [5–8, 13]. In other pulmonary diseases, anemia was shown to be a risk factor for increased duration of hospitalization and hospital admission [14]. However, Yang et al. [9] and Cecconi et al. [3] did not report a significant association between low Hb levels and COVID-19 survival. The observed discrepancy may be related to the design of the study (retrospective vs. prospective), sample size, and inclusion criteria.

The association between anemia and poor outcomes in unadjusted models may be partly related to higher age and higher prevalence of some comorbidities in anemic patients [15]. However, we also observed a significant association after accounting for the disease severity, presence of comorbidities, age, sex, and hypoxia status. This may be partly due to the effect of anemia on immunity, which in turn increases the probability of poor outcomes in patients with COVID-19 [16]. Furthermore, anemia activates the sympathetic nervous system, which increases heart rate, blood pressure, and pulmonary capillary leakage, causing acute respiratory distress syndrome (ARDS) [17].

The present study had some limitations. First, we defined anemia based on the levels of Hb on admission, and we had no information on the Hb levels before infection and dynamic Hb levels during hospitalization. Second, we did not measure other biomarkers of anemia, including serum iron, ferritin, and transferrin levels. Third, we included all patients in Imam Reza Hospital of Tabriz as a provincial and regional referral center for COVID-19, which might limit the generalizability of results.

The strengths of this study include the prospective nature of the study, including the large sample of patients with COVID-19, and considering a large number of confounding factors that may affect the association between anemia and COVID-19 outcomes.

## Conclusions

The results of the present study showed that the prevalence of anemia in hospitalized patients with COVID-19 was high, and it was associated with poor outcomes of COVID-19. From a practical point of view, the Hb level should be closely monitored during illness and hospitalization. However, further studies are required to confirm whether the Hb level can be used as a prognostic marker.

## Data Availability

The datasets generated and/or analyzed during the current study are not publicly available due to the institution’s policy, but are available from the corresponding author upon reasonable request.

## Abbreviations

ARDS: Acute respiratory distress syndrome
BMI: Body mass index
COVID-19: Coronavirus disease 2019
Curb-65: Confusion, urea, respiratory rate, blood pressure, and 65 years of age or older
DBP: Diastolic blood pressure
Hb: Hemoglobin
hs-CRP: High-sensitivity C-reactive protein
ICU: Intesive care unit
OR: Odds ratio
qSOFA: Quick sequential organ failure assessment
RR: Respiratory rate
RT-PCR: Reverse transcription-polymerase chain reaction
SARS-COV-2: Severe acute respiratory syndrome coronavirus
SBP: Systolic blood pressure
SD: Standard deviation
SPSS: Statistical package for social sciences
WBC: White blood cell
WHO: World health organization
